# LONELINESS AND SOCIAL ISOLATION AMONG VULNERABLE, DIVERSE, AND MINORITY GROUPS: NOVEL RESEARCH AND PRACTICAL INSIGHTS

**DOI:** 10.1093/geroni/igae098.0220

**Published:** 2024-12-31

**Authors:** Roger O’Sullivan, Christina Victor, Thomas Cudjoe

**Affiliations:** Chair; Co-Chair; Discussant

## Abstract

Loneliness and social isolation are global public health challenges with concerning implications for mental, cognitive, and physical health. Extensive evidence documents the prevalence, incidence, and negative health outcomes associated with loneliness and social isolation. In contrast, there is a dearth of research offering focused insights into three under-researched ‘at-risk’ populations: those with severe mental health illness, different racial and minority groups, and people who identify as LGBT. This symposium will address this gap by examining the experience of loneliness in these three populations and providing evidence to inform the development of future public health, policy, and service interventions. Pomeroy provides context with an overview of issues constraining progress in the field, including measurement inconsistency and the lack of diversity in studies of loneliness and social isolation. O’Sullivan, Taylor, and Victor highlight the importance of studying loneliness and social isolation within subpopulations and at-risk groups. O’Sullivan describes qualitative interviews with mental health patients to examine their conceptualization and lived experience of loneliness. Taylor’s paper examines the issue of race, neighborhoods and their confluence on feelings of loneliness among older adults in the United States. Victor using longitudinal data reports that rates of chronic loneliness over three years are twice as high amongst LGB and minority ethnic groups compared with the general population aged 50+. Cudjoe, as discussant, highlights the implications for policy, services, and future research in this area and the need for focused public health solutions to tackle loneliness and social isolation.

